# A Multi-Modal Deep-Learning Air Quality Prediction Method Based on Multi-Station Time-Series Data and Remote-Sensing Images: Case Study of Beijing and Tianjin

**DOI:** 10.3390/e26010091

**Published:** 2024-01-22

**Authors:** Hanzhong Xia, Xiaoxia Chen, Zhen Wang, Xinyi Chen, Fangyan Dong

**Affiliations:** 1 Faculty of Electrical Engineering and Computer Science, Ningbo University, Ningbo 315211, China; 2111082390@nbu.edu.cn (H.X.); 2211100281@nbu.edu.cn (Z.W.); 2 School of Mathematics and Statistics, Ningbo University, Ningbo 315211, China; 206000926@nbu.edu.cn; 3 Faculty of Mechanical Engineering and Mechanics, Ningbo University, Ningbo 315211, China

**Keywords:** air quality prediction, multi-modal data, remote-sensing image, graph neural network, deep learning, time-series prediction

## Abstract

The profound impacts of severe air pollution on human health, ecological balance, and economic stability are undeniable. Precise air quality forecasting stands as a crucial necessity, enabling governmental bodies and vulnerable communities to proactively take essential measures to reduce exposure to detrimental pollutants. Previous research has primarily focused on predicting air quality using only time-series data. However, the importance of remote-sensing image data has received limited attention. This paper proposes a new multi-modal deep-learning model, Res-GCN, which integrates high spatial resolution remote-sensing images and time-series air quality data from multiple stations to forecast future air quality. Res-GCN employs two deep-learning networks, one utilizing the residual network to extract hidden visual information from remote-sensing images, and another using a dynamic spatio-temporal graph convolution network to capture spatio-temporal information from time-series data. By extracting features from two different modalities, improved predictive performance can be achieved. To demonstrate the effectiveness of the proposed model, experiments were conducted on two real-world datasets. The results show that the Res-GCN model effectively extracts multi-modal features, significantly enhancing the accuracy of multi-step predictions. Compared to the best-performing baseline model, the multi-step prediction’s mean absolute error, root mean square error, and mean absolute percentage error increased by approximately 6%, 7%, and 7%, respectively.

## 1. Introduction

In the current scenario, the escalating frequency of severe air pollution, particularly in urban and metropolitan areas [1], has become a significant concern for citizens and society at large. The adverse impacts of air pollution extend beyond environmental degradation to encompass a spectrum of detrimental health consequences. PM_2.5_, also known as Particulate Matter 2.5, is a prevalent and highly dangerous air pollutant. It consists of tiny particles with a diameter of 2.5 micrometers or less, capable of lingering in the atmosphere for prolonged durations. Prolonged exposure to high concentrations of PM_2.5_ has been associated with increased incidence rates of asthma, cancer, respiratory diseases, and cardiovascular ailments, consequently elevating mortality rates [2,3]. Beyond its impact on human health, air pollution significantly affects the sustainable development of global socio-economic systems and ecosystems [4]. Given the crucial risk posed by air pollution, numerous nations have been implementing and reinforcing measures aimed at preventing air pollution [5]. Multiple monitoring stations have been established worldwide to track air pollutant emissions and gather real-time air quality data [6]. However, these monitoring stations provide solely current and past air quality data, making it challenging to undertake effective measures for preventing future air pollution. Accurately predicting air pollution based on historical data collected from multiple monitoring stations contributes significantly to identifying necessary preventive measures. This allows for avoiding exposure to air pollutants, safeguarding public health, and minimizing air pollution risks to the greatest extent possible. For instance, individuals can use air quality prediction results to determine whether a particular day is suitable for outdoor activities. Precise air pollution forecasting significantly aids urban sustainable development by assisting governments in implementing pollution control measures and formulating robust health policies.

The past decade has also witnessed the rapid advancement of remote-sensing technology, providing a wealth of multi-source remote-sensing data. The availability of these data has been continuously increasing, not only opening new possibilities for a better understanding of intelligent earth observations but also posing new methodological challenges in various remote-sensing applications, such as land-cover classification [7], mineral exploration, and mapping [8], scene understanding [9] and object detection [10]. These remote-sensing images also include observed geographical distribution data of PM_2.5_ [11,12], and we believe that remote-sensing images can serve as a new modularity source in modeling to improve the predictive performance of air quality prediction.

In addition, some works in image quality assessment (IQA) are also noteworthy. Wu et al. proposed a cascaded deep neural network based on CNNs for blind IQA, and this method effectively captures the representation of quality degradation [13]. Sun et al. introduced a distortion-graph representation learning framework GraphIQA for IQA, leveraging type-discriminative networks and blur prediction networks for blind image quality evaluation [14]. Liu et al. utilized a split-and-merge distillation strategy to train a single-headed network addressing lifelong blind IQA challenges [15]. Yang et al. presented a Transformer-based blind IQA with continual learning methods to enhance model transferability [16]. Su et al. introduced a dual-branch network to simultaneously learn low-level distortions and high-level semantics, employing masking label strategies and progressive weighted curriculums for IQA [17]. Saha et al. proposed an expert fusion approach for training two independent encoders to learn advanced content and low-level image quality features in an unsupervised environment, addressing the issue of automated perceptual IQA [18].

Conventional methods for air quality prediction primarily comprise interpretable statistical methods and machine learning models. Statistical methods, fundamentally grounded in mathematically interpretable models such as vector autoregressive (VAR) models [19] and autoregressive integrated moving average (ARIMA) [20], impose stringent requirements on input data, often necessitating data that pass stationarity tests. In contrast, machine learning methods do not demand specific input data prerequisites and adeptly address nonlinear fitting challenges. Ma et al. utilized the XGBoost method to forecast air quality in Shanghai [21]. Liu et al. employed intelligent algorithms to seek optimal parameters, proposing a genetic algorithm-based extreme learning machine model [22]. Patel et al. conducted sensitivity analysis to comprehend the individual factor impacts, subsequently employing a random forest model for predicting air quality in Delhi [23]. Ma et al. employed a variety of machine learning models such as ANN, XGBoost, and SVM to construct an ensemble method for predicting air quality in Macau [24]. However, due to the inherent limitations in model complexity, machine learning models often struggle to achieve optimal performance on big datasets.

Deep-learning methods possess superior feature extraction capabilities compared to traditional machine learning approaches. Most models expand their architectural structures or algorithms based on convolutional neural networks (CNNs) and recurrent neural networks (RNNs) to address the temporal variations in air quality. Yi et al. integrated various data sources, segregating them into spatial and temporal perspectives, and inputted the data into a deep neural network (DNN) [25]. Wang et al. achieved favorable outcomes in predicting air quality in Hebei Province, China, by utilizing the chi-square test algorithm and long short-term memory (LSTM) neural networks [26]. Li et al. employed bidirectional LSTM to model long-term dependency relationships within the data [27]. Jiang et al. opted for gated recurrent units (GRUs), a simplified framework of LSTM addressing gradient vanishing issues, for air quality prediction [28]. Wu et al. initially utilized variational mode decomposition to handle non-stationarity in air quality data and subsequently utilized LSTM for prediction [29]. Du et al. considered temporal context and proposed a bidirectional LSTM with an attention mechanism for multivariate time-series prediction [30]. Cheng et al. integrated ResNet and LSTM to forecast air quality, utilizing ResNet for spatial correlation extraction among stations and LSTM for capturing temporal dependencies [31]. Liang et al. combined various attention mechanisms with LSTM, employing an encoder–decoder architecture for multi-station air quality prediction [32]. Lin et al. utilized temporal convolutional networks (TCNs) alongside spatio-temporal attention to extract intricate dynamic features, further employing Bayesian optimization to explore optimal parameters [33]. Hu et al. employed Granger causality and k-means to identify strongly correlated stations, and subsequently used attention-LSTM to predict air quality [34]. However, these CNN- and RNN-based models excel in capturing temporal relationships while struggling to capture spatial correlations among different stations.

Subsequently, models based on graph neural networks (GNNs) emerged, effectively compensating for the limitations of CNNs in capturing spatial correlations between stations. Huang et al. used points of interest data in the city to construct topological structures, capturing spatial heterogeneity attributes [35]. Ge et al. leveraged points of interest data to establish latent geographical features and utilizing graph convolutional networks (GCNs) with TCNs to extract the spatio-temporal information [36]. Jin et al. utilized the propagation-based GNN GraphSAGE and GRU to extract spatio-temporal correlations, employing the Bayesian algorithm to search for optimal model hyperparameters [37]. Xiao et al. proposed a new similarity method by utilizing wind field data to build a bidirectional directed graph, employing GCN and GRU to develop a predictive model [38]. Wang et al. utilized diverse station interrelations to construct multiple graphs, embedding GCN within the encoder and decoder structures to predict air quality [39]. Chen et al. employ an attention mechanism and embedding layers to integrate multiple predefined graphs, utilizing GCN and TCN to capture complex spatio-temporal dependencies [40]. However, these models have all only utilized single-source time-series data and have not made use of remote-sensing image data.

The prediction of air pollutant concentrations has long been a focal point [41]. However, the variation in atmospheric pollutant concentrations is intricate and dynamic, spanning multiple sectors, regions, and dimensions of analysis [42]. Delving into the dynamic patterns of air pollutant concentration changes necessitates handling substantial volumes of data on air pollutant concentrations alongside pertinent meteorological information. Currently, there exists a substantial body of research in academia concerning the prediction of air quality. However, the predominant approach relies heavily on unimodal predictive methods, primarily involving the construction of machine learning or deep-learning models using historical time-series data such as air quality and meteorological data. We posit that leveraging remote-sensing image data to enrich the data sources and introduce new data modalities holds promise in enhancing the predictive efficacy of air quality prediction. Remote-sensing images offer additional visual information about pollutant dispersion, strengthening the predictive performance of the model.

In this paper, we place special emphasis on the demanding task of multi-station air quality prediction. This task has gained prominence due to its growing significance in areas such as precision urban planning, environmental monitoring, and cross-sectoral and societal governance. We present a multi-modal deep-learning model, Res-GCN, that concurrently processes time-series data from multiple stations and high-resolution remote-sensing images to predict future air quality. Specifically, we apply a residual neural network (ResNet) for extracting visual features from remote-sensing images and propose a dynamic spatio-temporal graph convolution network (DSTGCN) for capturing the spatio-temporal feature from time-series data. By fusing these two distinct modal features, the Res-GCN model is endowed with the capability to handle multi-source data. To validate the effectiveness of the Res-GCN model, experiments were conducted using the Beijing dataset, comparing it with several common mono-modal deep-learning models, along with ablation experiments. The main contributions of our work are summarized as follows:

1. We propose a new multi-modal deep-learning prediction model (Res-GCN) that utilizes ResNet to extract visual features from remote-sensing images and employs a dynamic spatio-temporal graph convolution network to extract spatio-temporal features from multi-station time-series data. By extracting two distinct modal features, Res-GCN achieves a more accurate prediction of air quality. To the best of our knowledge, we are the first to extract multi-modal features for prediction from both time-series data and remote-sensing images.
2. We introduce dynamic time pattern distance to construct dynamic graphs, better accommodating the temporal dynamics of air quality. The generated dynamic graphs, coupled with the dynamic graph convolutional network, aid in the flexible extraction of spatio-temporal features, thereby enhancing predictive performance.
3. Ablation experiments demonstrate the effectiveness of the components of Res-GCN. Comparative experiments reveal the superiority of Res-GCN over mono-modal models, affirming the utility of multi-modality.

The structure of this paper is outlined as follows: Section 2 elaborates on the problem definition of multi-station air quality prediction; Section 3 details the process of Res-GCN, a multi-modal prediction method based on ResNet and DSTGCN; Section 4 introduces the dataset and meticulously describes the experimental details and results; finally, the conclusions and prospects for future work are discussed in Section 5.

## 2. Problem Statement

In this paper, our aim is to predict future PM_2.5_ pollution concentrations across multiple monitoring stations utilizing historical time-series data and PM_2.5_ remote-sensing images. Within a time window *T*, the data from *N* monitoring stations are represented as $X=\{x_{1},x_{2},\cdots ,x_{N}\}$ , where $x_{n}\in R^{T\times F}$ denotes the *F*-dimensional features recorded by the *n*-th monitoring station. Furthermore, we construct a dynamic graph to aid in extracting features from the time-series data. This dynamic graph is constructed as an undirected graph denoted by $G=\left(V,E,A\right)$ , where *V* represents the set of nodes, each corresponding to a monitoring station. *E* signifies the edges connecting different nodes, and the relationships between nodes are represented by the adjacency matrix $A\in R^{N\times N}$ , where *N* is the number of nodes. Simultaneously, we incorporate the previous day’s remote-sensing image as auxiliary data in our modeling, denoted by $X_{R}\in R^{N_{L}\times N_{W}}$ , with $N_{L}$ indicating the image’s length and $N_{W}$ representing its width. It is important to note that these remote-sensing images contain solely single-channel information.

Utilizing historical time-series data *X*, the established graph *G*, and remote-sensing image $X_{R}$ as inputs, the aim of air quality prediction is to derive a function *f* that accurately forecasts future PM_2.5_ pollution concentrations $Y\in R^{N\times \tau \times 1}$ across *N* monitoring stations over a forthcoming duration of τ time steps.

(1) $$Y=f\left(X;X_{R};G\right)$$

## 3. Methodology

### 3.1. Overview

**Figure 1:**
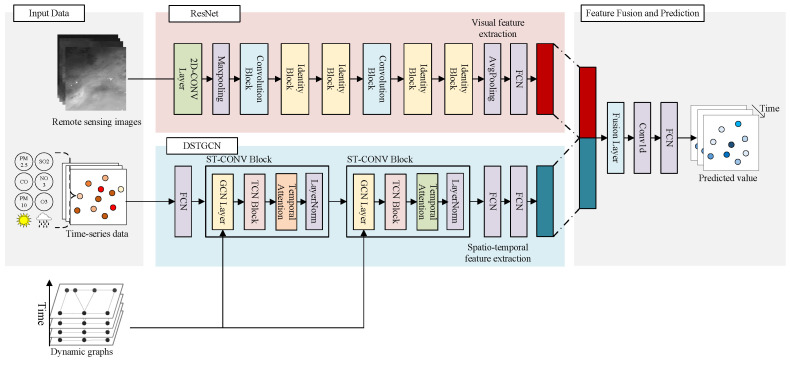
Overview of Res-GCN: FCN, fully connected layer; TCN, temporal convolution network; GCN, graph convolution network.

As illustrated in Figure 1, our proposed deep-learning model (Res-GCN) is intricately composed of a combination of residual networks (ResNets) and dynamic spatio-temporal graph convolutional networks (DSTGCNs). The primary objective of Res-GCN is to forecast the future air quality of multiple stations through the integration of features extracted from two different modalities of data. Our framework is delineated into three pivotal components: (1) We employ ResNet for the concealed extraction of visual features from remote-sensing images. These visual features encompass not only detailed information from images but also intricate patterns of pollution propagation; (2) Acknowledging the dynamic spatial correlation among stations, we calculate dynamic time warping (DTW) distances between every pair of stations to generate a dynamic graph. DSTGCN, in conjunction with the generated dynamic graph, extracts spatio-temporal features from the time-series data, thereby effectively capturing the dynamic relationships among stations; (3) We adopt a parameter matrix fusion approach to blend the extracted features from the two modalities. Ultimately, through a fully connected layer and a convolution layer, we amalgamate their outputs, generating predictions for future air quality at multiple stations. This fusion process contributes to the comprehensive utilization of both feature modalities, enhancing the predictive performance.

### 3.2. Residual Network

**Figure 2:**
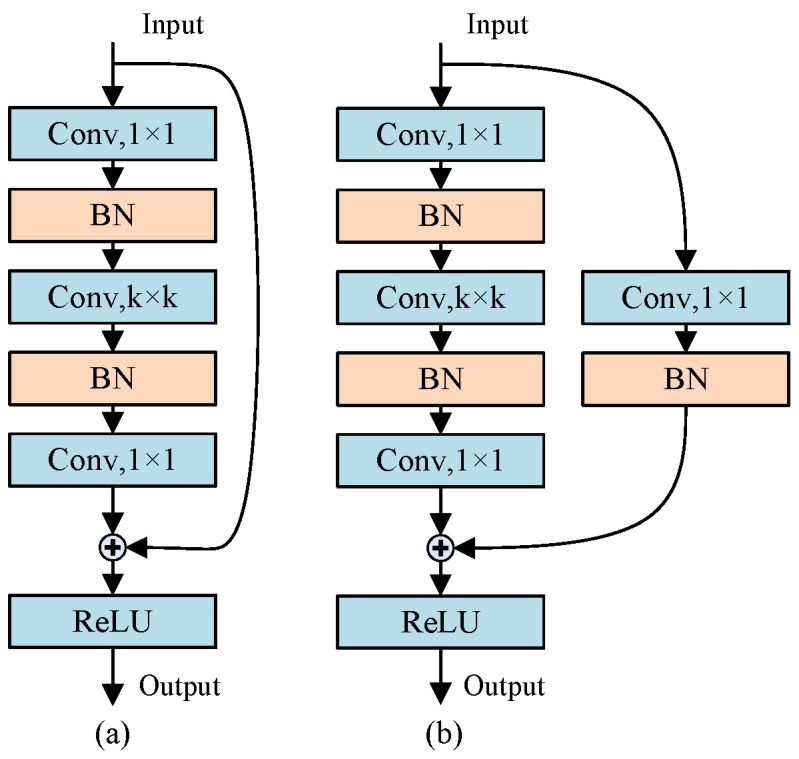
(**a**) Identity block in ResNet; (**b**) Convolution block in ResNet.

ResNet is employed for visual feature extraction from remote-sensing images. Introduced by He et al. [43], ResNet aims to facilitate feature learning within deep networks, resolving historical challenges associated with training deep models. The fundamental concept of ResNet involves establishing skip connections, referred to as residual blocks, between layers and their subsequent counterparts (as depicted in Figure 1) below. These residual blocks enable the network to capture latent information conducive to learning deeper hierarchical structures. To augment the capability for extracting profound features, contemporary ResNet models are typically designed with an emphasis on increased depth. However, due to the limited geographical scope of our remote-sensing dataset, primarily focusing on the Beijing region, we refrained from constructing an excessively deep ResNet. Our ResNet architecture, illustrated in Figure 1 below, comprises a convolutional layer for dimension embedding, multiple residual blocks for feature extraction, two pooling layers, and two fully connected layers for dimension alignment. From Figure 2, we adopted two primary types of residual blocks: identity blocks and convolutional blocks. The identity blocks reduce the number of trainable parameters through parameter-free identity shortcuts, while the convolutional blocks employ one-dimensional convolutions for dimension matching. To mitigate the parameter count during training, both types of residual blocks are integrated into our architecture. Each residual block consists of three conventional 2D convolutional layers utilized for feature extraction and batch normalization layers to ensure smooth gradient propagation. The kernel sizes within the convolutional layers are, respectively, 1×1 , 3×3 , and 1×1 .

### 3.3. Dynamic Spatio-Temporal Graph Convolution Network

Considering the significant modal disparities between multi-station time-series data and remote-sensing images, the approach to feature extraction should also diverge. We propose a dynamic spatio-temporal graph convolution network (DSTGCN) for extracting spatio-temporal features from time-series data, positioning DSTGCN as an advancement of STGCN. As depicted in Figure 1 below, DSTGCN comprises multiple spatio-temporal convolutional blocks. Each block includes a graph convolutional layer for capturing spatial features among stations, a temporal convolutional block for extracting long-range temporal dependencies, and a temporal attention mechanism to redistribute weights, emphasizing crucial time steps. Diverging from STGCN, which utilizes station latitude and longitude distances to construct a static graph, DSTGCN employs DTW distance for constructing a dynamic graph based on time series within the time window. This choice better accommodates variations in pollutant diffusion scenarios.

#### 3.3.1. Dynamic Graph Construction

The spatial correlation between stations is highly dynamic, and the dispersion of air pollutants varies under different meteorological conditions. Therefore, relying solely on static graphs makes it challenging to capture the intricate dynamic spatial correlations. We utilize sparse dynamic time warping (DTW) distance to construct dynamic graphs. DTW, referenced in [44], stands as a comprehensively adopted approach to gauge the similarity of two time series. This method involves facilitating warped alignment and calculating distances between time points across two time series to ascertain their resemblance. Let $T_{i}=\left\{T_{i}^{1},T_{i}^{2},\ldots ,T_{i}^{m}\right\}$ and $T_{j}=\left\{T_{j}^{1},T_{j}^{2},\ldots ,T_{j}^{m}\right\}$ represent two time series for station *i* and station *j*, each comprising *m* time steps. Here, $T_{i}^{t}$ and $T_{j}^{t}$ denote two points at time step *t* in the respective time series. The DTW method calculates the Euclidean distance between every pair of time points in $T_{i}$ and $T_{j}$ , resulting in an accumulated distance matrix represented as *d*. Subsequently, it updates the warping matrix D using the following equation:

(2) $$D\left(T_{i}^{t},T_{j}^{t}\right)=d\left(T_{i}^{t},T_{j}^{t}\right)+\min\left\{\begin{array}{c} D(T_{i}^{t-1},T_{j}^{t})\\ D(T_{i}^{t-1},T_{j}^{t-1})\\ D(T_{i}^{t},T_{j}^{t-1}) \end{array}\right.$$

Derived from the warped path W , the optimal path is computed to establish a sequence of neighboring matrix entries matching between $T_{i}$ and $T_{j}$ . This path aims to minimize the overall distance between $T_{i}$ and $T_{j}$ . Denoted as $W=\{W_{1},W_{2},\ldots ,W_{K}\}$ , with *K* being the count of elements in W and following the condition $\max(|T_{i}\left|,\right|T_{j}\left|\right)\leq K\leq \min(|T_{i}\left|,\right|T_{j}\left|\right)$ , DTW seeks the optimal path amidst numerous possibilities to minimize the overall distance. The DTW distance formula between two time series, $T_{i}$ and $T_{j}$ , is expressed as

(3) $$DTW\left(T_{i},T_{j}\right)=\min_{W}\left[\frac{1}{K}\sqrt{\sum_{k=1}^{K}W_{k}}\right]$$

**Figure 3:**
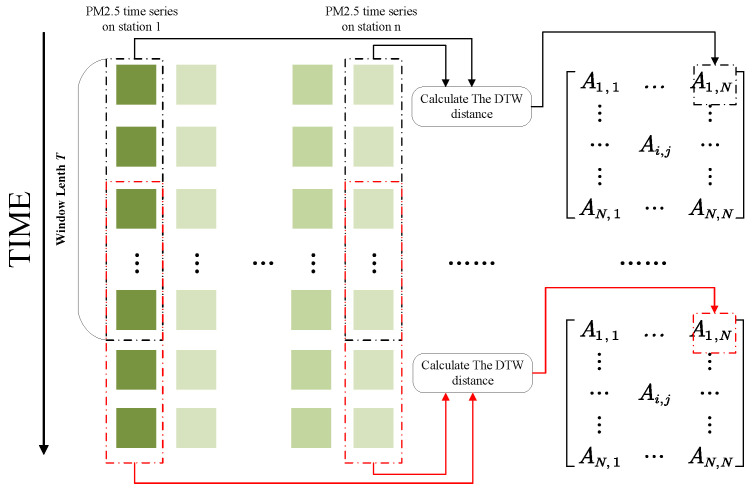
Utilizing DTW distance to compute the similarity between two PM_2.5_ time series for the generation of the matrix element of dynamic graph (illustrated by $A_{1,N}$ ).

As shown in Figure 3, we utilize the DTW distance among PM_2.5_ time series from various monitoring stations to create the dynamic graph’s adjacency matrix. It is crucial to highlight that these time series are segmented into time windows, enabling the construction of adjacency matrices across distinct time intervals. This method improves the model’s ability to adjust to the ever-changing temporal patterns in time-series data. The graph construction formula employing DTW distance is outlined as follows:

(4) $$A=\left[\begin{array}{ccc} A_{1,1} & \cdots & A_{1,N}\\ \vdots & \; & \vdots \\ \cdots & A_{i,j} & \cdots \\ \vdots & \; & \vdots \\ A_{N,1} & \cdots & A_{N,N} \end{array}\right]$$

(5) $$A_{i,j}=\left\{\begin{array}{cc} \exp\left(-\frac{DTW{\left(x_{i}^{PM_{2.5}},x_{j}^{PM_{2.5}}\right)}^{2}}{\varphi^{2}}\right), & \exp\left(-\frac{DTW{\left(x_{i}^{PM_{2.5}},x_{j}^{PM_{2.5}}\right)}^{2}}{\varphi^{2}}\right)\geq \epsilon \\ 0, & \;\mathrm{Otherwise}\; \end{array}\right.$$

where $A_{i,j}$ is the element in the *i*-th row and *j*-th column of the adjacency matrix *A*; $x_{i}^{PM_{2.5}}$ and $x_{j}^{PM_{2.5}}$ , respectively, denote the PM_2.5_ time series of monitoring station *i* and monitoring station *j*; $DTW(x_{i}^{PM_{2.5}},x_{j}^{PM_{2.5}})$ is the DTW distance between the PM_2.5_ sequence of stations *i* and *j*; φ is the variance of the Gaussian kernel; and ϵ is the threshold value. It is noteworthy that the dynamic graph adjacency matrix generated for each time window is distinct. The adjacency matrix utilized for subsequent GCN layers to extract spatial features undergoes variations as the time window slides.

#### 3.3.2. Graph Convolutional Network Layer

Traditional CNNs excel in handling regularized features such as images, videos, and audio features because these characteristics can be represented in a grid-like format. However, the distribution of real spatial stations comprises irregular, non-Euclidean spatial graphs, making it difficult for traditional CNN models to capture the underlying spatial dependencies among monitoring stations. To tackle this issue, the graph convolutional network (GCN) layer is introduced to process data structured in graphs, extending CNNs from their traditional use with data organized in regular grids to data organized in graph structures. We opt to utilize GCN to capture the implicit spatial dependencies among stations.

Given the adjacency matrix of dynamic graph $A\in R^{N\times N}$ and the graph signal matrix $H^{\left(l\right)}\in R^{T\times N\times F^{\left(l\right)}}$ , GCN constructs convolutional filters in the spectral domain to capture spatial features from *A* and *H*. These convolutional filters aggregate first-order neighboring information of each node to capture spatial dependencies between nodes. GCN can stack multiple convolutional filters to aggregate more information from higher-order neighborhoods, and this process can be represented as:

(6) $$H^{\left(l+1\right)}=GCN\left(H^{\left(l\right)}\right)=ReLU\left({\tilde{D}}^{-\frac{1}{2}}\tilde{A}{\tilde{D}}^{-\frac{1}{2}}H^{\left(l\right)}W^{\left(l\right)}\right)$$

where $F^{\left(l\right)}$ represents the number of features in the graph signal, $W^{\left(l\right)}\in R^{F^{\left(l\right)}\times F^{\left(l+1\right)}}$ is the trainable weight matrix of the *l*-th layer, $\tilde{A}=A+I_{N}$ is the adjacency matrix with self-connections, ${\tilde{D}}_{ii}=\sum_{j=1}^{N}{\tilde{A}}_{ij}$ is the degree matrix, and ReLU is the activation function.

#### 3.3.3. Temporal Convolution Network Block

**Figure 4:**
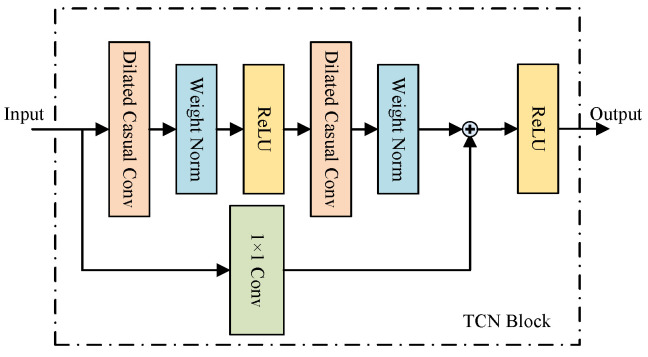
Details of TCN block.

**Figure 5:**
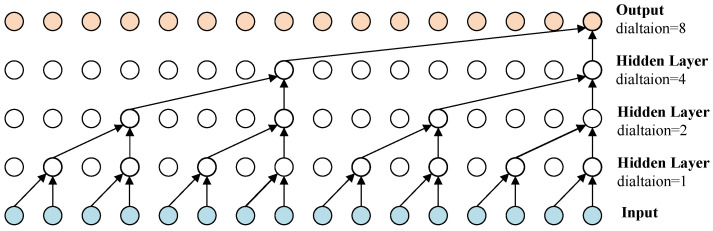
Example of casual dilation convolution with dilation rate 2, 4, 6, 8.

After conducting graph convolution operations to capture the neighboring information of each node in the spatial dimension of the graph, we employ a TCN block to capture correlations and update the node signals. TCN employs convolutional operations to process time-series data, utilizing sliding windows of convolutional kernels to capture patterns and relationships across different time steps within the sequence. This parallelized architecture facilitates quicker training of TCN and enables more effective handling of lengthy sequences without relying on recurrent structures [45]. As depicted in Figure 4, the TCN block comprises two causal dilated convolutional layers designed to capture long-range temporal dependencies. Additionally, it includes two weight normalization layers to prevent overfitting and introduces the ReLU activation function to incorporate non-linearity. It also contains a shortcut to ensure smooth gradient propagation. As shown in Figure 5, causal dilation convolution is a variant of the standard one-dimensional convolution. Instead of convolving all elements, it employs a dilation strategy, skipping some elements to achieve a larger receptive field. For a univariate input sequence *i* in a single dimension, the dilated convolution with kernel *w* can be expressed as:

(7) $$\begin{array}{cc} {\mathrm{Conv}}_{K_{t}}^{\mathrm{dil}}\left(i\right)=\left(i{*}_{dil}w\right)\left(t\right) & =\sum_{k_{t}=0}^{K_{t}-1}w\left(k_{t}\right)i\left(t-dil\cdot k\right) \end{array}$$

where $K_{t}$ is the kernel size, dil is the dilation rate, and i(t) is the *t*-th element of input sequence. Employing multiple dilated convolutions allows networks to possess extensive receptive fields, capturing long-range temporal dependencies while maintaining a reduced number of layers. Specifically, as the quantity of stacked layers grows, the network’s depth also increases. The TCN block can be expressed as follows:

(8) $$\begin{array}{cc} TCN(H) & =\mathrm{WN}\left({\mathrm{Conv}}_{K_{t}}^{\mathrm{dil}}\left(ReLU\left(\mathrm{WN}\left({\mathrm{Conv}}_{K_{t}}^{\mathrm{dil}}\left(H\right)\right)\right)\right)\right)+{\mathrm{Conv}}_{1\times 1}\left(H\right) \end{array}$$

where *H* denotes the input of the TCN block, WN denotes the weight normalization layer, and ${\mathrm{Conv}}_{1\times 1}$ denotes the 1×1 convolution layer.

#### 3.3.4. Temporal Attention

The temporal attention mechanism is a frequently utilized technique tailored for time-series data processing. Its fundamental concept revolves around integrating a mechanism within the model to emphasize crucial information across various time steps. We employed the temporal attention mechanism to capture dependencies between time steps and redistribute weights to pivotal time steps. This mechanism allows the model to concentrate on the most pertinent time steps, and its formulation can be articulated as follows:

(9) $$\begin{array}{c} E=V_{e}ReLU\left(\left({\left(H\right)}^{T}W_{1}\right)W_{2}{\left(\left(H\right)W_{3}\right)}^{T}+b_{e}\right)\\ E_{i,j}^{\prime }=\frac{\exp\left(E_{i,j}\right)}{\sum_{j=1}^{T}\exp\left(E_{i,j}\right)}\\ \mathrm{Tatt}\left(H\right)=E^{\prime }H \end{array}$$

where *H* is the input feature matrix; $W_{1}$ , $W_{2}$ , and $W_{3}$ are the trainable weight matrices; $V_{e}$ is the trainable weight vector; $b_{e}$ is the trainable bias vector; *E* is the attention matrix; $E^{\prime }$ is the normalized attention matrix; and Tatt is the temporal attention function.

#### 3.3.5. Spatio-Temporal Convolutional Block

To integrate spatial and temporal features, we employ a spatio-temporal convolutional block (STConv block) when handling time-series data, combined with a constructed dynamic graph for processing. The STConv block can be stacked or expanded based on the specific scale and complexity; in this paper, we opted for a two-layer stack. As illustrated in Figure 1 below, each STConv block comprises a graph convolutional layer, a TCN block, and a temporal attention mechanism, effectively extracting latent spatio-temporal features. Additionally, layer normalization is applied within each STConv block to prevent overfitting. The formula for the STConv block can be expressed as:

(10) $$\begin{array}{cc} STConv(H) & =\mathrm{LN}\left(\mathrm{TATT}\left(\mathrm{TCN}\left(\mathrm{GCN}\left(H\right)\right)\right)\right) \end{array}$$

where *H* denotes the input of the STConv block, and LN denotes the layer normalization layer. After two layers of ST-CONV blocks, convolutional layers and fully connected layers are employed for dimension alignment, ensuring consistency in the dimensions of the two modalities for subsequent fusion.

### 3.4. Feature Fusion and Prediction

After extracting the visual features and spatio-temporal features using ResNet and DSTGCN, respectively, a method based on parameter matrices is applied to merge the outputs of the aforementioned models [46]. The process of the fusion layer can be described as follows:

(11) $$\begin{array}{c} z=\sigma \left(H_{ST}W_{ST}+H_{V}W_{V}+b_{z\;\;}\right)\\ H_{F}=z⊙H_{ST}+\left(1-z\right)⊙H_{V} \end{array}$$

where $H_{ST}$ and $H_{V}$ are the outputs of DSTGCN and ResNet, respectively; $W_{ST}$ and $W_{V}$ are the trainable weight matrices of DSTGCN and ResNet, respectively; $b_{z}$ is the trainable bias vector. σ denotes the sigmoid function; ⊙ denotes the element-wise product; and $H_{F}$ is the fused feature matrix. The resultant fused feature matrix $H_{F}$ is then processed through a fully connected layer and a 1D convolution layer to produce the final prediction results.

(12) $$\begin{array}{c} \hat{Y}=\mathrm{FC}\left(ReLU\left(\mathrm{Conv}\left(H_{F}\right)\right)\right) \end{array}$$

where $\hat{Y}$ is the predicted value of the air quality data, FC denotes the fully connected layer, Conv denotes the 1D convolution layer, and ReLU is the activation function.

### 3.5. Loss Function

The loss function serves to quantify the disparity between the predicted and actual values, playing a pivotal role in the model’s efficacy. Its selection significantly impacts the model’s performance. In this study, we employ the mean squared error (MSE) as our chosen loss function, defined by the following formula:

(13) $$\begin{array}{c} \mathrm{MSE}=\frac{1}{N_{s}}\sum_{i=1}^{N_{s}}{\left({\hat{y}}_{i}-y_{i}\right)}^{2} \end{array}$$

where $N_{s}$ denotes the count of training samples, ${\hat{y}}_{i}$ is the predicted value of the *i*-th training sample, and $y_{i}$ is the actual value of the *i*-th training sample.

## 4. Experiments and Results

### 4.1. Dataset

#### 4.1.1. Time-Series Dataset

**Figure 6:**
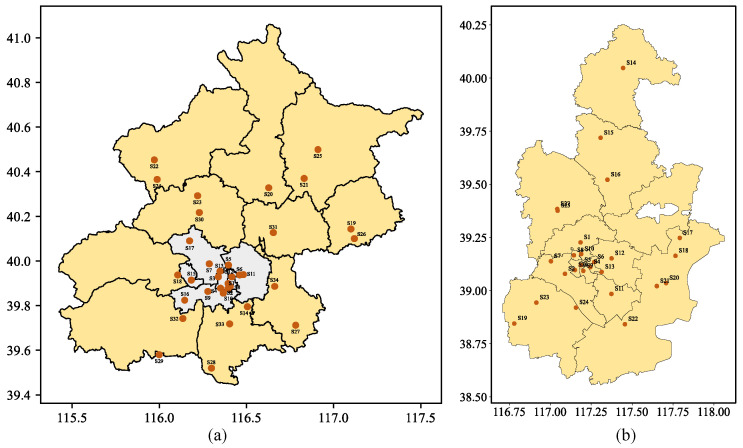
(**a**) Geographical distribution of 34 monitoring stations in the Beijing dataset. The white areas denote the main urban areas of Beijing; (**b**) Geographical distribution of 27 monitoring stations in the Tianjin dataset.

This paper selects Beijing and Tianjin in China, as the focal cities to evaluate the model’s predictive performance regarding PM_2.5_ pollutant concentration. The time-series data comprise two categories: air quality data and meteorological data. The air quality data are provided by the China National Monitoring Center (http://www.cnmc.cn/ssj/ accessed on 1 September 2023), while the meteorological data originate from the National Climatic Data Center (https://www.ncdc.noaa.gov/NCDC accessed on 1 September 2023). The air quality data for Beijing comprise historical hourly records from 34 national air quality monitoring stations spanning from 1 January 2018 to 1 January 2021, encompassing a total of 26,280 time steps. For Tianjin, the air quality data include historical hourly records from 27 air quality monitoring stations covering the period from 1 May 2014 to 1 May 2015, amounting to a total of 8760 time steps. Each monitoring station records six hourly air pollutant concentrations (PM_2.5_, PM_10_, SO_2_, NO_2_, CO, and O_3_). These monitoring stations are distributed across various districts within Beijing and Tianjin, as illustrated in Figure 6. The meteorological data comprise four hourly factors (temperature, air pressure, dew point temperature, and wind speed). It is worth noting that the temporal resolution of time-series data is hourly.

Owing to issues such as sensor malfunctions or equipment maintenance at monitoring stations, the time-series data across various monitoring stations exhibit missing values. In the Beijing dataset, there are approximately 924 missing time steps, accounting for approximately 4% of the total data volume. Meanwhile, the Tianjin dataset exhibits around 633 missing time steps, constituting approximately 6% of the total data volume. While handling missing values within the dataset pertaining to atmospheric pollutant concentrations and meteorological factors, if data for a single time step are absent, the data from the preceding time step are utilized for imputation. In instances of continuous missing data spanning multiple time steps, assuming a uniform trend within this period, arithmetic sequence data are employed for filling in the gaps. The remaining missing values are addressed using linear interpolation. Eventually, all missing values are adequately filled.

The Beijing dataset is partitioned into training, validation, and testing sets in a ratio of 7:1:2. The data were partitioned based on chronological order. The specific temporal ranges are as follows: the training set spans from 1 January 2018 to 5 February 2021, the validation set ranges from 6 February 2021 to 23 April 2021, and the test set covers the period from 24 April 2021 to 31 December 2021. The Tianjin dataset is partitioned into training, validation, and testing sets in a ratio of 8:1:1. The specific temporal ranges are delineated as follows: the training set spans from 1 May 2014 to 17 February 2015, the validation set covers the period from 18 February 2021 to 25 March 2021, and the test set encompasses the timeframe from 26 March 2021 to 1 May 2015. To standardize the data and facilitate quicker model convergence, we apply Z-score normalization to scale the dataset.

#### 4.1.2. Remote-Sensing Images’ Dataset

In this paper, the remote-sensing images are sourced from ChinaHighPM_2.5_ [11,12]. ChinaHighPM_2.5_ encapsulates high-resolution, fully covered, high-quality, and long-term remote-sensing images of ground-level air pollutants in China, collectively known as ChinaHighAirPollutants (CHAP). The CHAP dataset comprises seamless (100% spatial coverage) 1-km ground-level PM_2.5_ remote-sensing images on a daily, monthly, and yearly basis, spanning from 2000 to 2021.

**Figure 7:**
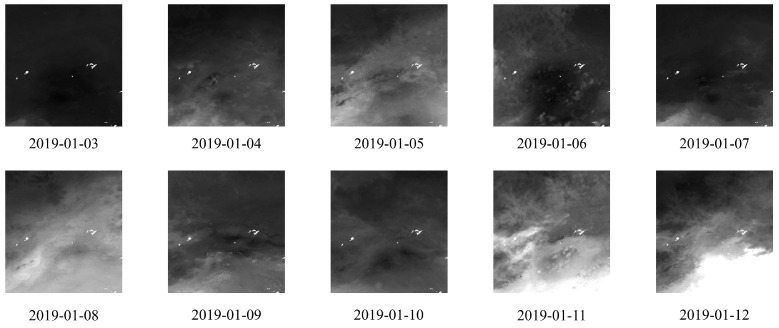
Samples of processed remote-sensing images for Beijing.

**Figure 8:**
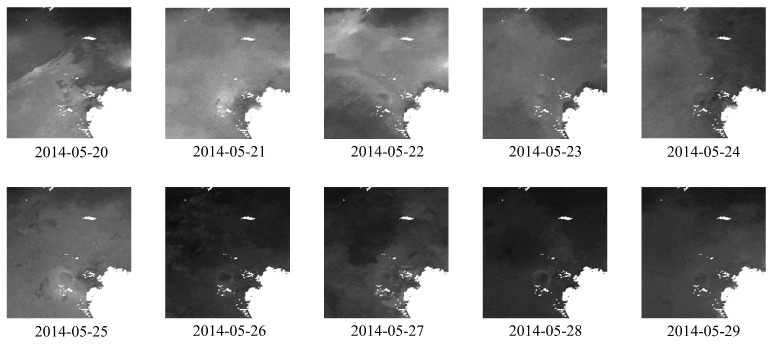
Samples of processed remote-sensing images for Tianjin.

For the Beijing dataset, we curated a set of images collected daily between 2018 and 2021 to serve as inputs for the model. For the Tianjin dataset, we opted for images acquired daily from 1 May 2014 to 1 May 2015, for model input. As the original remote-sensing images are in NetCDF format, we initially utilized Python programs to convert the format into TIFF image files. Subsequently, we performed geographic cropping based on the coordinates of Beijing and Tianjin (Beijing’s longitude ranging from 115.7° E to 117.4° E, and latitude from 39.4° N to 41.6° N; Tianjin’s longitude ranging from 116.3° E to 118.2° E, and latitude from 38.5° N to 40.3° N), resulting in images with a resolution of 220×210 and 210×200 pixels. To facilitate the model input, we conducted central cropping, image normalization, and removal of redundant image channels from the processed images. These operations were carried out to prepare the images with a resolution of 210×210 and 200×200 for the model input. The samples of remote-sensing images from Beijing and Tianjin are illustrated in Figure 7 and Figure 8.

### 4.2. Evaluation Criteria

To assess the predictive error of the proposed model, this paper employs two commonly used error metrics for time-series forecasting: root mean square error (RMSE), mean absolute error (MAE), and mean absolute percentage error (MAPE). The formulas for these three metrics are as follows:

(14) $$\begin{array}{c} \mathrm{RMSE}=\sqrt{\frac{1}{N_{s}}\sum_{i=1}^{N}{\left({\hat{y}}_{i}-y_{i}\right)}^{2}}\\ \mathrm{MAE}=\frac{1}{N_{s}}\sum_{i=1}^{N}\left|{\hat{y}}_{i}-y_{i}\right|\\ \mathrm{MAPE}=\frac{1}{N_{s}}\sum_{i=1}^{N}\left|\frac{{\hat{y}}_{i}-y_{i}}{y_{i}}\right| \end{array}$$

where $N_{s}$ is the number of samples, ${\hat{y}}_{i}$ is the predicted value of the *i*-th sample, and $y_{i}$ is the actual value of the *i*-th sample.

### 4.3. Model Parameter Configurations

In the experiment, we utilized the data from the preceding 24 h (*T* = 24) to forecast future PM_2.5_ pollutant concentration for the subsequent 24 h ( τ=24 ). The input of the model also encompassed remote-sensing images, specifically cropped images from the day before the prediction date, with a resolution of 210×210 or 200×200 pixels. In ResNet, we extensively employed 3×3 and 1×1 two-dimensional convolutional layers, culminating in the utilization of an average pooling layer with a size of 34 (set to 27 during the training on the Tianjin dataset). In DSTGCN, we employ the ChebyshevGCN layer [47] for spatial feature extraction, setting the graph convolution order K=2 . Furthermore, in the TCN block, we set the dilation rates for dilated convolutions to 2 and 4. Temporal attention was implemented through a multi-headed attention mechanism with eight attention heads. Regarding the model’s training strategy, we configured the learning rate and batch size to 0.001 and 32, respectively. To prevent model overfitting, an early stopping strategy was adopted; training ceased if the validation loss did not decrease for ten consecutive iterations. L2 regularization with a weight of 0.001 was introduced into the loss function. Additionally, a dropout layer was employed with a parameter of 0.3. The Adam optimizer was chosen to optimize the weights of the model. The maximum iterations were set to 100, and the best-performing model weights were selected based on validation loss. PyTorch 1.12 framework was employed for model training. Table 1 shows the detailed hardware configurations.

### 4.4. Performance Comparison

To showcase the predictive performance of the proposed Res-GCN model, seven baseline models were selected for comparison. All baseline models were trained and tested using identical training, validation, and testing time-series datasets. Detailed information regarding the selected baseline models is provided below:

1. ARIMA [20]: Autoregressive integrated moving average model, comprehensively used as an interpretable statistical model for time-series forecasting.
2. SVR [48]: Support vector regression model, a machine learning model that utilizes support vectors for regression tasks.
3. DNN: Deep neural network, a basic deep-learning model that consists of multiple densely fully connected layers with ReLU activation function.
4. LSTM [49]: Long short-term memory model, an RNN variant extensively utilized for processing and learning from time-series data.
5. CNN-LSTM [50]: A combined model using CNN for handling spatial features and LSTM for capturing temporal characteristics.
6. TCN [45]: Temporal convolutional network, primarily composed of stacked dilated convolutional layers and residual blocks. Compared to LSTM, it excels in capturing long-range temporal dependencies.
7. STGCN [51]: Specifically designed GNN model for spatio-temporal graph prediction. It leverages GCN and temporal gate convolution to capture hidden spatio-temporal correlations.
8. Informer [52]: A Transformer-based model for time-series forecasting. It utilizes a prob-sparse self-attention mechanism to capture long-range temporal dependencies.
9. STSGCN [53]: A spatio-temporal graph prediction model based on graph neural networks. It combines local graphs from multiple time steps to construct a large synchronized graph, utilizing GCN to extract spatio-temporal dependencies from the synchronized graph.

Five training runs were conducted for both the Res-GCN and baseline models, and the average test set metrics for each method were reported, as depicted in Table 2. Specifically, Table 2 illustrates the MAE, RMSE, and MAPE metrics for the prediction of future PM_2.5_ air pollutant concentrations at 1-h, 8-h, 16-h, and 24-h horizons by the Res-GCN model and other baseline models. In most cases, the proposed Res-GCN outperformed the baseline model across the three evaluation metrics. Notably, DNN exhibited approximately 10% lower MAE compared to SVR, indicating that deep-learning methods generally outperform statistical and machine-learning methods (ARIMA and SVR). Moreover, LSTM demonstrated superior predictive performance compared to DNN, suggesting that RNN models can better capture temporal characteristics compared to models solely comprising densely fully connected layers. Models combining multiple methods, such as CNN-LSTM, displayed enhanced predictive capabilities, outperforming single models like LSTM and TCN by effectively capturing multivariate features. As the forecasting horizon increased, STGCN and Res-GCN exhibited relatively smaller fluctuations in MAE and RMSE errors compared to ARIMA, SVR, DNN, and LSTM. Informer leverages a probability-sparse self-attention mechanism to enhance the Transformer architecture. In comparison to STGCN, it exhibits superior predictive performance. STSGCN employs multiple local graphs to construct a comprehensive synchronous graph, thereby achieving a larger spatio-temporal receptive field. In contrast to STGCN, it demonstrates superior spatio-temporal capture capabilities. The observation underscores the impact of GNN-based models in predicting, indicating their capability to capture spatial correlations among monitoring stations and demonstrating efficacy in long-term forecasting. Furthermore, leveraging additional modal data from remote-sensing images, Res-GCN acquired richer visual features pertaining to finer-scale pollutant dispersion. Irrespective of the forecasting horizon, it consistently achieved the lowest error.

**Figure 9:**
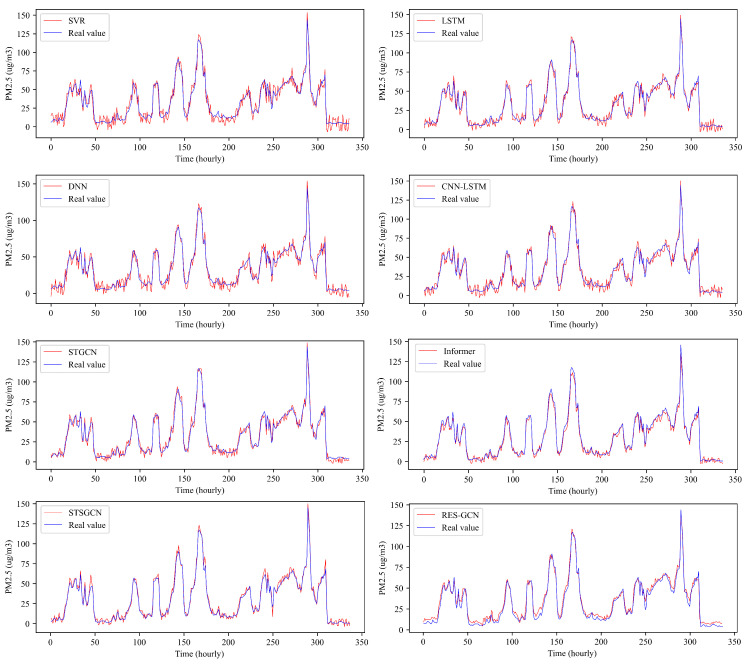
One-hour predictions and actual PM_2.5_ values for different models on Beijing dataset.

We conducted visualization of PM_2.5_ prediction curves for the S3 Guanyuan station in Beijing over the forthcoming two-week period using the aforementioned baselines (excluding ARIMA). As illustrated in Figure 9, nearly all models proficiently captured the trend of the one-hour prediction task time series. However, models like DNN and LSTM displayed oscillations in their prediction curves, notably deviating from the actual curve. Conversely, CNN-LSTM and STGCN effectively captured the trend while maintaining errors within a reasonable range. Among the models, STGCN and Res-GCN appeared more effective compared to CNN and RNN-based models, possibly due to the utilization of GCN networks, which efficiently extract spatial characteristics among the stations. Res-GCN demonstrated superiority in trend prediction, exhibiting lower errors and achieving a balance between trend prediction and minimizing deviation from the actual values.

### 4.5. Model Component Analysis

In this section, we conducted ablation experiments on two datasets to evaluate the effectiveness of the ResNet and DSTGCN components within the model. ResNet is utilized to extract relevant visual features from remote-sensing images, while DSTGCN focuses on extracting impactful spatio-temporal features from time-series data. We chose to remove ResNet (w/o ResNet) and DSTGCN (w/o DSTGCN) from the Res-GCN model, creating two variants. The w/o ResNet variant represents the scenario where the model lacks input from remote-sensing images, while the w/o DSTGCN variant signifies the absence of input from time-series data. This experiment also validates the contributions of the two data sources to the model’s performance. Table 3 presents the performance of Res-GCN and its variants in predicting PM_2.5_ air pollutant concentrations at different temporal scales. From the results, a certain decline in predictive performance is observed upon removing ResNet, emphasizing the crucial role of augmented remote-sensing image data as a data source in model building. Remote-sensing images encapsulate rich visual information that effectively assists the model in capturing additional details, thereby enhancing predictive performance. Conversely, removing DSTGCN results in a noticeable deterioration in predictive performance. This underscores the pivotal role of time-series data in multi-station air quality prediction tasks. Without time-series data, the model struggles to grasp the fundamental spatio-temporal feature crucial for precise air quality predictions. The role of remote-sensing images primarily serves as an auxiliary in capturing more latent visual information. This visual information includes more fine-grained details of pollutant dispersion, effectively aiding the model in enhancing predictive performance. In conclusion, the strategic use of ResNet for extracting visual information from remote-sensing image data, coupled with the utilization of DSTGCN for capturing spatio-temporal dependencies in air quality data, constitutes an effective design for enhancing short-term and long-term prediction capabilities.

### 4.6. Different Graph Construction Methods’ Comparison

In this section, a performance comparison is conducted between different methods used to construct the graph adjacency matrix. Specifically, we selected the employment of Euclidean distance, Pearson correlation, Spearman correlation, and DTW distance for graph construction and compared their respective performances. The comparison of the graph construction methods, specifically DTW against the other methods, is presented in Table 4. It is evident that utilizing DTW distance for graph construction outperforms other methods in predicting PM_2.5_ air pollutant concentrations across various ranges. Particularly noteworthy is the method employing the geographical coordinates of the stations, which exhibited the poorest performance. This can be attributed to the geographical station construction method solely considering fixed geographic information while neglecting the intricate trend variations within the time series. Consequently, the constructed graph struggled to capture dynamism. Conversely, the utilization of DTW distance for graph construction proved superior to the two correlation-based methods. This indirectly suggests that DTW distance, to some extent, encapsulates the similarity between the lengths of the two time series. In contrast, the two correlation methods only address the linear correlation between features, resulting in a similarity measure that does not emphasize dynamic temporal patterns. The results indicate that encoding dynamic spatial relations between stations using DTW distance is effective and can enhance the short-term and long-term predictive capabilities for improving air quality.

**Figure 10:**
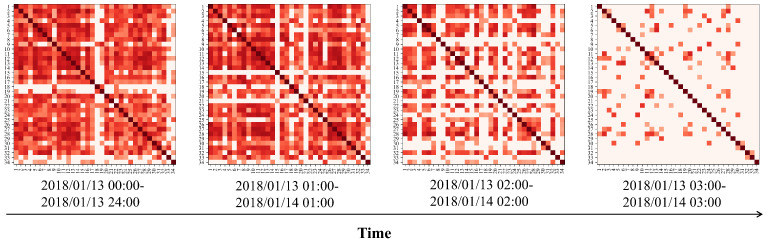
Heat map visualization of the dynamic graph adjacency matrices generated by using DTW distance on Beijing dataset.

**Figure 11:**
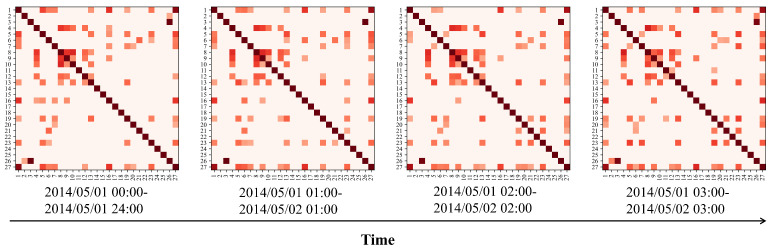
Heat map visualization of the dynamic graph adjacency matrices generated by using DTW distance on Tianjin dataset.

As shown in Figure 10 and Figure 11, we visualized the dynamic graphs generated by DTW distance. It can be observed that selecting different time windows for time series results in different dynamic graphs. This ensures that the generated graph matrix can more flexibly adapt to the GCN layer to capture dynamic spatial features.

### 4.7. Multi-Station Prediction Performance

**Figure 12:**
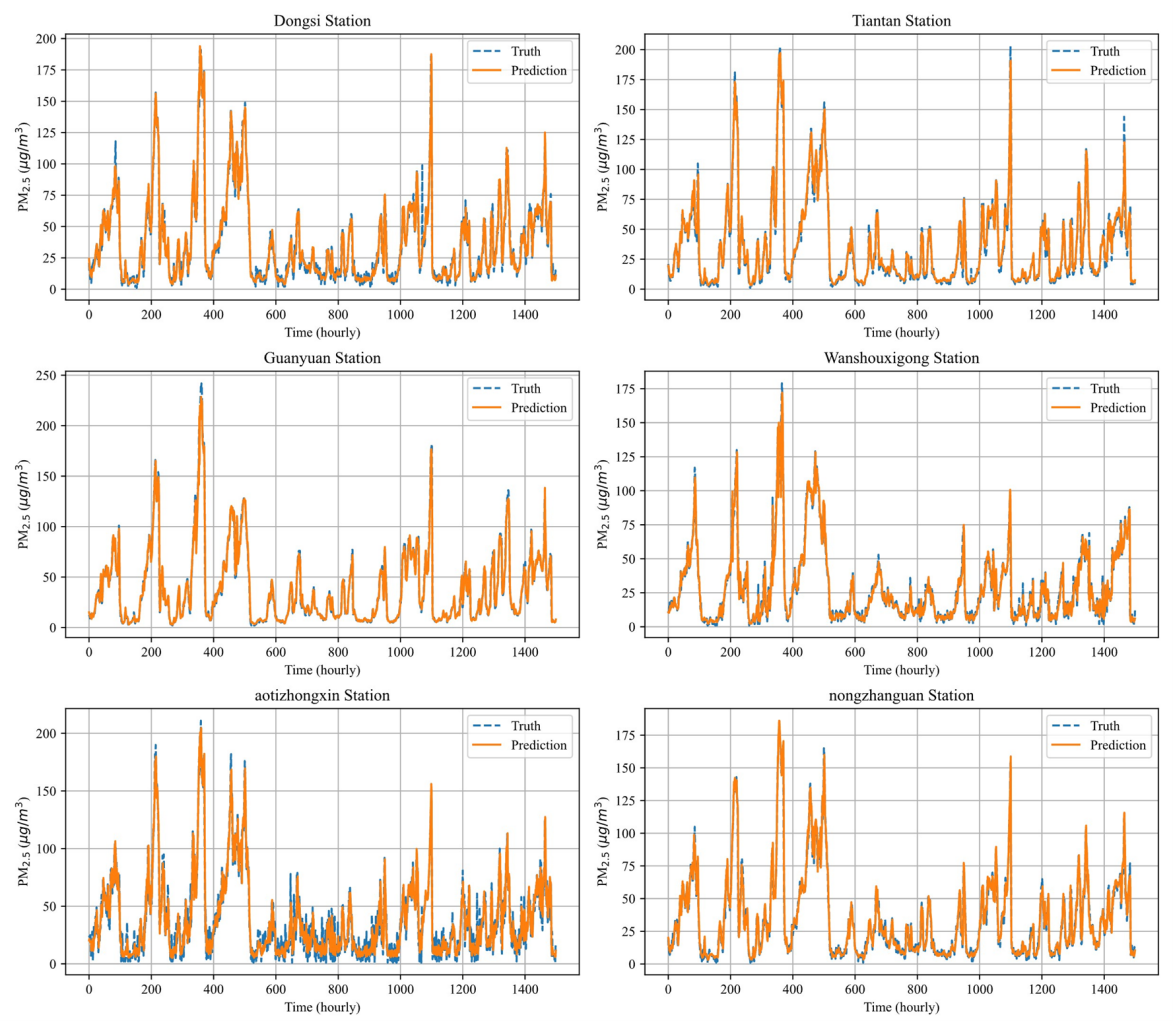
Visualization of one-hour PM_2.5_ concentration prediction curve for multiple stations in the Beijing dataset.

**Figure 13:**
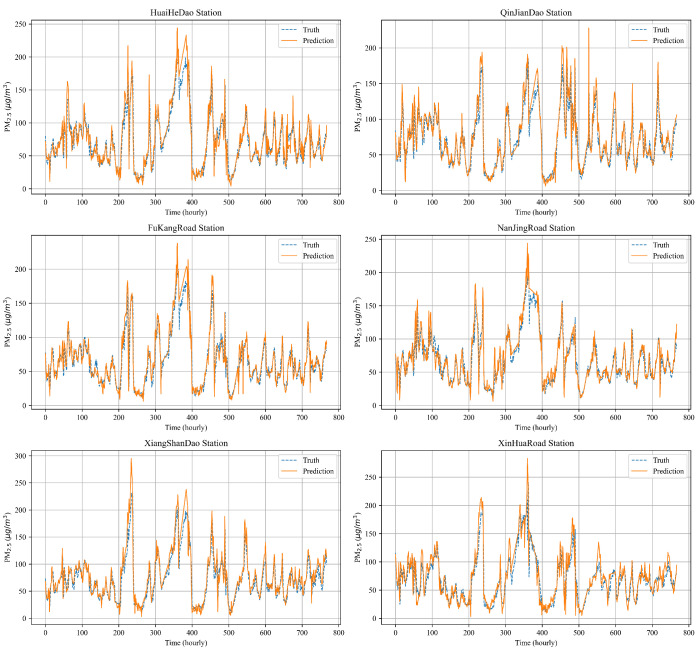
Visualization of one-hour PM_2.5_ concentration prediction curve for multiple stations in the Tianjin dataset.

Figure 12 depicts the predicted PM_2.5_ concentration curves at six monitoring stations in Beijing (S12 Dongsi, S8 Tiantan, S3 Guanyuan, S4 Wanshouxigong, S5 Aotizhongxing, S6 Nongzhanguan) over a one-hour period. Figure 13 illustrates the predicted PM_2.5_ concentration curves at six monitoring stations in Tianjin (S1 HuaiHeDao, S8 QinJianDao, S3 FuKangRoad, S5 NanJingRoad, S6 XiangShanDao, S7 XinHuaRoad) over a one-hour period. Res-GCN demonstrates outstanding generalization abilities in forecasting complex and non-stationary PM_2.5_ air pollutant data. This suggests that the model is not restricted to specific stations or data types but can adapt to various data patterns. The model performs exceptionally well in individual station prediction tasks and equally achieves significant advancements in multi-station air quality prediction. This underscores Res-GCN’s capability to integrate multi-station data and achieve accurate predictions, providing substantial support for precise air quality forecasting within urban areas.

## 5. Conclusions

In this paper, we present a new multi-modal prediction model named Res-GCN, designed to improve the accuracy of air quality predictions by integrating multiple modalities of data, including multi-station time-series data and remote-sensing images. Res-GCN consists of two key components: ResNet and DSTGCN. Primarily, ResNet effectively extracts representative visual features of fine-grained pollutant dispersion in remote-sensing images through the stacking of residual blocks. This enables the model to comprehensively understand and articulate the visual information pertaining to the impact of air quality. To capture dynamic spatio-temporal dependencies in time-series data, DSTGCN employs DTW distance to generate dynamic graphs, offering a flexible encoding of spatial correlations between monitoring stations. By integrating graph Convolutional networks (GCNs) and temporal convolutional networks (TCN) with temporal attention, DSTGCN effectively extracts spatio-temporal features from the time-series data. In summary, Res-GCN, by simultaneously capturing two different modalities of data, namely multisite time-series data and image data, can comprehensively learn the complex and dynamic relationships between air pollutants and their surrounding environment. The experimental results on two real-world datasets indicate that Res-GCN exhibits significant improvements, ranging from 30% to 40%, in predicting short-term and long-term air quality data compared to statistical and machine learning models such as ARIMA and SVR. In contrast to traditional deep-learning models (DNN, LSTM, TCN, CNN-LSTM), Res-GCN demonstrates a performance enhancement of 20–30% in predictive accuracy. Moreover, when compared to machine learning models, Res-GCN outperforms them. In comparison to the graph network model STGCN, Res-GCN achieves a performance increase ranging from 6% to 15%. Additionally, when compared to the best baseline models (Informer, STSGCN), Res-GCN shows a 5–6% improvement. In comparison to mono-modal models, Res-GCN significantly improves performance, providing more accurate information for the prevention and management of air pollutants. By notifying citizens and communities about potential air quality risks, Res-GCN shows potential for enhancing public health outcomes.

In the future, we will further explore the potential of Res-GCN in other fields, such as traffic prediction and urban planning. Additionally, we will explore the possibility of integrating other data, such as points of interest, to further enhance the predictive performance of Res-GCN.

## Figures and Tables

**Table 1:** Hardware configurations used in experiment.

Environment	Configuration
Operating system	Ubuntu 22.04 OS
CPU	Intel i5-13600 KF 3.5 GHZ
GPU	NVIDIA RTX4080 16 G
RAM	32 G
Hard disk	SN-850 1 TB
Python version	3.9.12

**Table 2:** Predictive performance of Res-GCN and other baselines on two datasets.

	τ=1			τ=8			τ=16			τ=24		
Beijing	MAE	RMSE	MAPE	MAE	RMSE	MAPE	MAE	RMSE	MAPE	MAE	RMSE	MAPE
ARIMA	13.22	19.12	0.40	17.88	25.58	0.63	22.33	31.22	0.86	25.55	35.55	1.34
SVR	11.48	16.22	0.37	15.98	23.88	0.57	20.22	28.36	0.83	23.24	34.22	1.28
DNN	10.22	14.02	0.36	14.58	22.28	0.54	18.02	25.55	0.78	21.44	30.12	1.25
LSTM	7.32	11.02	0.34	13.12	19.24	0.52	16.06	21.89	0.76	18.23	26.1	1.22
TCN	6.96	10.48	0.32	12.02	18.28	0.50	15.55	21.02	0.74	17.68	25.22	1.18
CNN-LSTM	6.88	10.52	0.28	11.24	17.02	0.46	14.88	20.23	0.70	16.56	23.78	1.18
STGCN	6.22	9.56	0.27	10.98	15.22	0.45	14.22	19.84	0.68	16.24	23.65	1.04
Informer	5.32	8.23	0.29	10.22	14.22	0.44	14.02	19.64	0.64	16.12	23.72	1.02
STSGCN	5.63	8.66	0.26	10.34	14.31	0.44	13.88	19.22	0.63	15.33	22.12	0.96
Res-GCN	5.30	7.80	0.24	9.54	14.05	0.42	13.25	18.19	0.61	15.22	20.97	0.93
Tianjin	MAE	RMSE	MAPE	MAE	RMSE	MAPE	MAE	RMSE	MAPE	MAE	RMSE	MAPE
ARIMA	19.16	28.12	0.42	27.22	35.62	0.57	33.34	40.22	0.80	36.55	45.25	1.07
SVR	16.68	26.34	0.38	25.66	34.55	0.53	31.56	38.32	0.76	34.46	43.22	0.98
DNN	15.44	25.46	0.36	24.57	33.12	0.51	31.02	37.55	0.68	33.24	42.14	0.94
LSTM	14.77	22.72	0.36	23.28	32.02	0.54	28.22	35.49	0.65	30.23	40.12	0.88
TCN	14.67	22.62	0.37	22.88	31.56	0.51	27.84	34.89	0.64	29.47	38.80	0.84
CNN-LSTM	14.44	22.12	0.35	22.46	30.11	0.49	26.58	33.78	0.62	29.02	38.56	0.82
STGCN	13.12	21.12	0.32	21.58	29.11	0.49	25.22	33.42	0.61	28.34	38.06	0.84
Informer	11.92	19.12	0.31	21.22	28.84	0.50	24.88	33.11	0.62	27.99	38.02	0.78
STSGCN	11.12	19.26	0.24	20.42	28.33	0.45	24.02	32.88	0.59	27.12	37.56	0.72
Res-GCN	11.44	19.02	0.24	19.88	28.05	0.44	23.43	31.82	0.58	26.99	36.88	0.70

**Table 3:** Predictive performance of Res-GCN and its variants on two datasets.

	τ=1			τ=8			τ=16			τ=24		
Beijing	MAE	RMSE	MAPE	MAE	RMSE	MAPE	MAE	RMSE	MAPE	MAE	RMSE	MAPE
w/o DSTGCN	11.22	17.76	0.38	14.24	22.12	0.60	21.92	27.78	0.81	25.64	34.45	1.12
w/o ResNet	5.99	8.88	0.27	10.33	14.84	0.46	14.02	19.23	0.65	16.22	22.22	0.97
Res-GCN	5.30	7.80	0.24	9.54	14.05	0.42	13.25	18.19	0.61	15.22	20.97	0.93
Tianjin	MAE	RMSE	MAPE	MAE	RMSE	MAPE	MAE	RMSE	MAPE	MAE	RMSE	MAPE
w/o DSTGCN	27.88	38.88	0.42	30.24	45.23	0.62	36.92	49.78	0.73	41.64	55.45	0.94
w/o ResNet	12.22	21.34	0.25	21.02	29.88	0.48	25.24	33.23	0.61	28.83	38.96	0.74
Res-GCN	11.44	19.02	0.24	19.88	28.05	0.44	23.43	31.82	0.58	26.99	36.88	0.70

**Table 4:** Comparison of different graph construction methods on two datasets.

	τ=1			τ=8			τ=16			τ=24		
Beijing	MAE	RMSE	MAPE	MAE	RMSE	MAPE	MAE	RMSE	MAPE	MAE	RMSE	MAPE
Euclidean distance (static)	5.88	9.12	0.27	9.99	14.84	0.45	13.66	18.76	0.65	15.46	22.02	0.97
Pearson	5.66	8.98	0.26	9.82	14.28	0.43	13.32	18.44	0.63	15.22	21.44	0.95
Spearman	5.72	8.77	0.26	9.77	14.22	0.44	13.34	18.56	0.63	15.34	21.76	0.94
SDTW	5.30	7.80	0.24	9.54	14.05	0.42	13.25	18.19	0.61	15.22	20.97	0.93
Tianjin	MAE	RMSE	MAPE	MAE	RMSE	MAPE	MAE	RMSE	MAPE	MAE	RMSE	MAPE
Euclidean distance (static)	12.02	20.23	0.26	21.10	29.02	0.47	24.22	33.07	0.61	28.18	38.12	0.74
Pearson	11.64	19.53	0.25	20.16	28.45	0.46	24.02	32.48	0.59	27.42	37.54	0.73
Spearman	11.67	19.42	0.25	20.12	28.66	0.45	23.89	32.56	0.59	27.44	37.66	0.72
SDTW	11.44	19.02	0.24	19.88	28.05	0.44	23.43	31.82	0.58	26.99	36.88	0.70

## Data Availability

The data are available upon request from the corresponding author.
